# Characterization of potential biomarkers of reactogenicity of licensed antiviral vaccines: randomized controlled clinical trials conducted by the BIOVACSAFE consortium

**DOI:** 10.1038/s41598-019-56994-8

**Published:** 2019-12-30

**Authors:** January Weiner, David J. M. Lewis, Jeroen Maertzdorf, Hans-Joachim Mollenkopf, Caroline Bodinham, Kat Pizzoferro, Catherine Linley, Aldona Greenwood, Alberto Mantovani, Barbara Bottazzi, Philippe Denoel, Geert Leroux-Roels, Kent E. Kester, Ingileif Jonsdottir, Robert van den Berg, Stefan H. E. Kaufmann, Giuseppe Del Giudice

**Affiliations:** 1 Max Planck Institute for Infection Biology (MPIIB), Department for Immunology, Berlin, Germany; 2 Present Address: Core Unit Bioinformatics, Berlin Institute of Health, Berlin, Germany; 3 Surrey Clinical Research Centre, University of Surrey, Guildford, UK; 4 Humanitas Clinical and Research Center-IRCCS, Rozzano, Italy; 5 Humanitas University, Pieve Emanuele, Italy; 6 The William Harvey Research Institute, Queen Mary University of London, London, UK; 7 GSK, Rixensart, Belgium; 8 Center for Vaccinology, Ghent University and University Hospital, Ghent, Belgium; 9 Sanofi-Pasteur, Swiftwater, PA USA; 10 deCODE genetics/Amgen Inc., Reykjavik, Iceland; 11 GSK, Rockville, MD USA; 12 GSK, Siena, Italy

**Keywords:** Vaccines, Predictive markers

## Abstract

Biomarkers predictive of inflammatory events post-vaccination could accelerate vaccine development. Within the BIOVACSAFE framework, we conducted three identically designed, placebo-controlled inpatient/outpatient clinical studies (NCT01765413/NCT01771354/NCT01771367). Six antiviral vaccination strategies were evaluated to generate training data-sets of pre-/post-vaccination vital signs, blood changes and whole-blood gene transcripts, and to identify putative biomarkers of early inflammation/reactogenicity that could guide the design of subsequent focused confirmatory studies. Healthy adults (N = 123; 20–21/group) received one immunization at Day (D)0. Alum-adjuvanted hepatitis B vaccine elicited vital signs and inflammatory (CRP/innate cells) responses that were similar between primed/naive vaccinees, and low-level gene responses. MF59-adjuvanted trivalent influenza vaccine (ATIV) induced distinct physiological (temperature/heart rate/reactogenicity) response-patterns not seen with non-adjuvanted TIV or with the other vaccines. ATIV also elicited robust early (D1) activation of IFN-related genes (associated with serum IP-10 levels) and innate-cell-related genes, and changes in monocyte/neutrophil/lymphocyte counts, while TIV elicited similar but lower responses. Due to viral replication kinetics, innate gene activation by live yellow-fever or varicella-zoster virus (YFV/VZV) vaccines was more suspended, with early IFN-associated responses in naïve YFV-vaccine recipients but not in primed VZV-vaccine recipients. Inflammatory responses (physiological/serum markers, innate-signaling transcripts) are therefore a function of the vaccine type/composition and presence/absence of immune memory. The data reported here have guided the design of confirmatory Phase IV trials using ATIV to provide tools to identify inflammatory or reactogenicity biomarkers.

## Introduction

Early clinical evaluations of vaccine and/or adjuvant safety focus on common well-characterized reactogenicity events, such as injection site pain, but may not allow prediction of low-incident safety events, which can then emerge during larger late-phase trials^1,2^. The immune mechanisms underlying reactogenicity may be unraveled in preclinical or clinical systems vaccinology studies^3,4^, though so far, extrapolation to later-stage clinical studies and standardization have been lacking. There is thus a need to develop reliable biomarkers of vaccine safety that are directly correlated with the occurrence of infrequent post-vaccination events in later-phase studies, or during post-licensure safety monitoring. Such markers could render these late phases more efficacious and cost-efficient, and could in early development phases help to prioritize candidates and avoid later failure of vaccines due to safety issues.

Supported by the Innovative Medicine Initiative-Joint Undertaking^5^, which fosters public-private partnerships between various academic groups and pharmaceutical companies in Europe, the public-private partnership BIOVACSAFE (Biomarkers for enhanced vaccines immunosafety; www.biovacsafe.eu) was created. This consortium was the first systematic approach of systems vaccinology to provide information on vaccine safety. The multi-phased project aimed to first identify, and then translate biomarkers of vaccine/adjuvant safety into practical tools for use in different development phases^6^. In the first clinical project phase, reported here, we administered relevant licensed vaccines with a well-known and extensive clinical safety/reactogenicity profile to healthy adults (20/21 per treatment group), in order to generate a training data-set for parameters considered to be reliable predictors of early inflammation or reactogenicity. The objective was to identify putative biomarkers of inflammation or reactogenicity that could guide the design of follow-on large scale non-residential confirmatory studies using selected vaccines and time points. Combined with preclinical studies conducted in parallel^7–10^, this data-set informed the design of subsequent clinical research phases aiming to confirm or refute the validity of these putative biomarkers. In this work, we applied an integrated systems biology approach based on the analysis of high-throughput data combined with detailed clinical and laboratory parameters^11^.

Though often dismissed as not clinically significant in clinical trials, several behavioral factors, including diet, exercise or fluid intake, have been identified as potentially affecting physiological and immune functions^12–14^. In this project, we performed identically designed, placebo-controlled clinical studies in the controlled in-patient setting of a chronobiology research centre, followed by one month of outpatient follow-up. This way, it was possible to eliminate this source of baseline variability, and to employ an intensive clinical monitoring and blood sampling schedule in the critical first days following immunization. This allowed us to capture the subtle short-term physiological changes/reactogenicity expected to be induced by these safe vaccines, and to harness this data with early inflammatory mediators, including changes in transcriptional expression and cytokine/chemokine responses detected in blood. The studies employed a panel of prototypical antiviral vaccination strategies and enrolled either naive or primed subjects. This enabled head-to-head comparison of the effects of different vaccine types and formulations (subunit with/without adjuvant, or live-attenuated), vaccination regimens (prime-boost or single-dose), and immune priming statuses.

We previously reported limited data from these clinical studies, focusing on specific topics, such as post-vaccination neutropenia and different aspects of the adaptive (Treg, T_FH_) responses to these vaccines^15–17^. In the present paper, we report the vital signs data, blood changes and, importantly, the gene transcripts detected in whole blood before and after vaccination, as generated in these clinical trials.

## Results

In saline placebo-controlled studies, we evaluated live yellow fever virus (YFV) and varicella zoster virus (VZV) vaccines in YFV-naive and VZV-primed subjects respectively (Study A); a prime-boost-boost regimen with aluminum-hydroxide (alum)-adjuvanted subunit hepatitis B virus (HBV) surface antigen vaccine in initially HBV-naive subjects (Study B); and non-adjuvanted or MF59-adjuvanted subunit trivalent influenza vaccine (TIV or ATIV respectively) in subjects assumed to have been exposed previously to circulating seasonal influenza viruses (Study C); see Table 1. Vaccines or placebo were administered at Day (D)0 during the inpatient stay (D-1 ─ D5; see Fig. S1 for detailed study designs). Subjects of Study B (referred to as the HBV-1 group up to D28) also received a booster dose at D28 during the outpatient follow-up, and a third dose at D169, during their second inpatient stay (the HBV-3 group).

**Table 1:** Demographics and reactogenicity.

Study	A		B		C		ABC
Group	VZV	YFV	HBV-1	HBV-3	ATIV	TIV	Placebo
Vaccine	Live	Live	Subunit	Subunit	Subunit	Subunit	NA
Adjuvant	none	none	Alum	Alum	MF59C.1	none	NA
Immunity at pre	+	−	−	+	+	+	NA
N vaccinated	21	20	21	20	20	21	20
Age in years Mean (min-max)	25 (19–44)	26.5 (21–42)	31 (20–45)		27.5 (20–43)	24 (18–39)	29.5 (20–42)
Ethnicity (A:B:O:W ratio)	2:2:1:16	4:0:3:13	2:2:2:15		0:1:0:19	0:3:1:17	2:1:1:16
Gender (F:M ratio)	7:14	7:13	4:17		10:10	11:10	9:11
Total number of AEs recorded^a^	10	14	17	0	30	10	8
Proportion of sub-jects with any AE	0.3	0.2	0.4	0.0	0.9	0.5	0.3
Reactosum^b^	64	27	70	0	84	24	17
Mean reactosum^b^ per subject	3.2	1.4	3.3	0.0	4.2	1.1	0.8

Abbreviations: NA, not applicable. Immunity at pre, presence (+) or absence (−) of immunity against the vaccine antigen(s) at pre-vaccination (baseline). A:B:O:W ratio, self-reported ethnicity (Asian:Black:Other:White) ratio. F:M ratio, female-to-male ratio. AEs, adverse events. aSome participants recorded more than one AE, or none. bReactosum denotes the sum of all reactogenicity scores by vaccine group, with reactogenicity scores calculated as: severity grade x duration [days] of treatment-related AEs with an onset between Day 0 and Day 7.

### Reactogenicity

Local and systemic AEs were collected during the inpatient and outpatient phases based on queries in which no symptoms of interest were specifically solicited. As expected, mostly mild reactogenicity was observed for these licensed vaccines, with the most common events being fever, injection site pain and headache. To achieve a uniform reactogenicity quantification across the groups that could serve as a basis for the correlative analyses in the subsequent studies, we calculated an integrated metric for all unsolicited local and systemic AEs per group. This was performed by summing (by subject and by day) the total recorded severity gradings across all treatment-related unsolicited local and systemic AEs, and calculating the overall group scores (Table 1). The scores obtained for the ATIV group were considered the most informative for future use in systems biology analyses.

### Vital signs, hematology, and blood biochemistry

**Figure 1:**
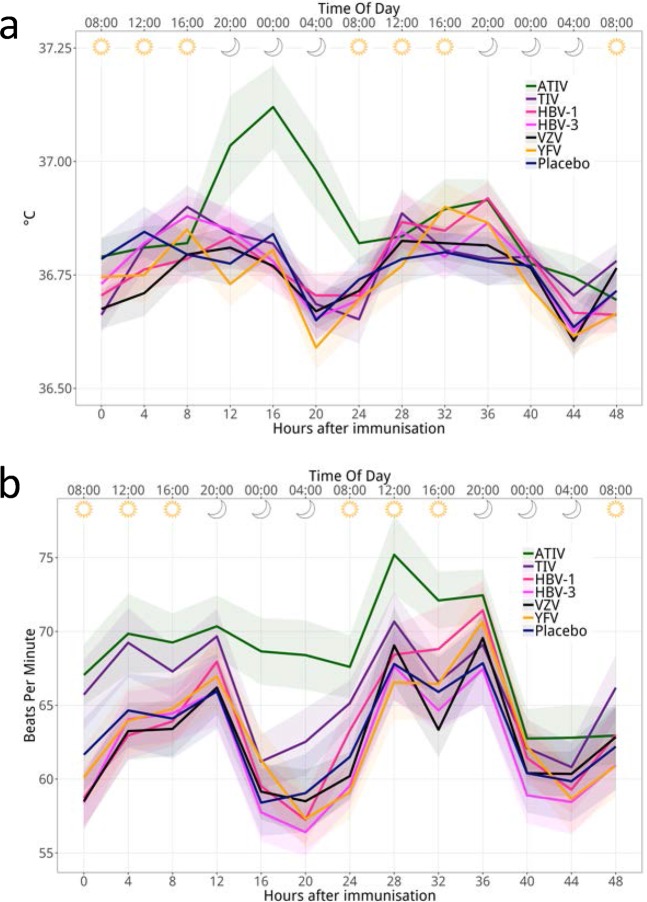
Oral temperature and pulse measurements after immunisation. The figure shows the group mean oral temperature (**a**), and pulse rate (**b**), recorded at various time points during the first 48 hours post vaccination for each treatment group. Ribbons indicate the SEM by group.

Four-hourly recording of vital signs during the inpatient stay revealed the expected diurnal changes, with subtle decreases in the oral body temperature and heart rate (~0.2 °C and ~5 bpm) during the first two nights post-vaccination, which were seen in all groups except the ATIV group (Fig. 1). Remarkably, the ATIV group exhibited in the first night (16–24 hours post-dose) a transient rise in baseline temperature (~0.3 °C), which remained within the normal range and was also not accompanied by a decreased nocturnal heart rate. As this was not observed with TIV, the data suggest that the effect was mediated by the oil-in-water (o/w) adjuvant MF59. This effect would not have been detected in conventional vaccine trials, which are typically not performed in inpatient settings and do therefore not allow for highly synchronized, nocturnal measurements.

Kinetics in WBC, lymphocyte, neutrophil and monocyte counts varied between vaccine types, with the most prominent patterns seen in the ATIV and YFV groups (Fig. S2). In the ATIV group, the transient increases in monocytes and neutrophils at D1 concurred with a transient decrease in lymphoid cells (+1.4 and −0.7 fold-change [FC], respectively). No clear differences between the inpatient and outpatient phases were identified, except that there was an increase of neutrophils (0.2 ─ 0.3 FC) following discharge from the residential unit across the treatment groups, including the placebo group. This increase was typically associated with adverse events of intercurrent infections in some subjects, as shown by an increased variance in the neutrophil count FCs in those groups (Fig. S2).

**Figure 2:**
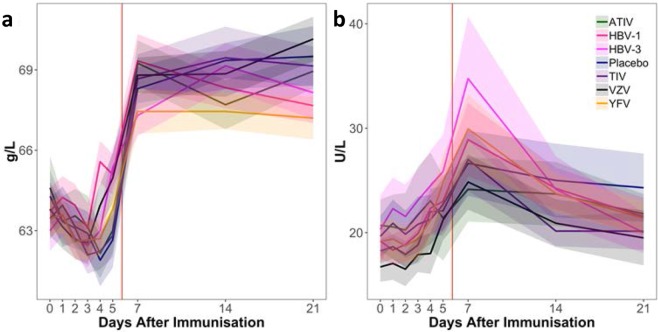
Changes in serum total protein and alanine transaminase concentrations post-immunization. The figure shows the mean concentrations of serum total protein (**a**; normal range 60–80 g/L) and alanine transaminase (**b**; normal range 0–49 U/L), measured on selected days after immunisation on Day 0. Ribbons indicate group SEM. Vertical red lines indicate the time of discharge from the residential unit.

In all vaccine or placebo groups, plasma levels of total protein showed a slight and gradual decline (<5%) during the inpatient stay, followed by a marked (10%) increase between D5 (after which participants left the inpatient unit) and the first outpatient visit on D7, to reach levels that remained constant up to D28 (Fig. 2a). This was also seen with albumin and with uncorrected calcium levels, as may be expected (Table S1). Alanine transaminase levels remained constant up to D5 in all groups, but increased by 10–50% between D5 and D7, during the immediate period after discharge from the residential unit (Fig. 2b). Thereafter, levels gradually decreased up to D28 in all vaccine groups (although only slightly in the ATIV group) but not in the placebo group. While all vaccine groups exhibited similar levels of total protein and alanine transaminase in the inpatient phase, the intergroup variation in both parameters increased substantially in the outpatient phase. Both parameters were thus clearly impacted by the changes in (inpatient/outpatient) trial setting, given that the changes at D5–7 were also seen in the controls.

### Acute-phase proteins, and serum cytokines/chemokines

**Figure 3:**
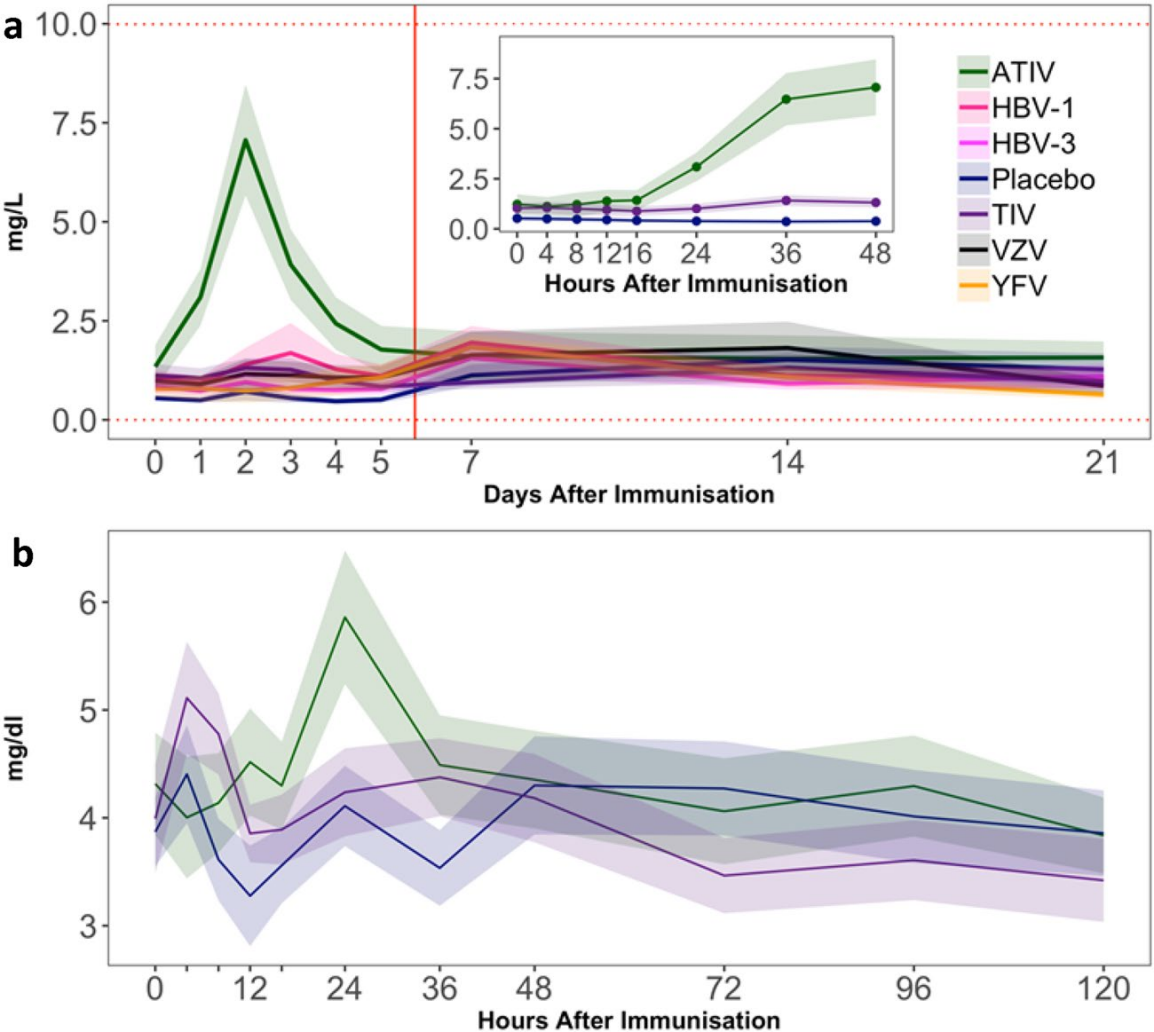
Changes in serum CRP and PTX3 concentrations post-immunization. Mean concentrations of C-Reactive Protein (CRP) and pentraxin 3 (PTX3) were measured on selected days after immunisation on Day 0. Panel (a) Normal CRP range (0–10 mg/L) is indicated by horizontal dotted red lines. Inset panel: CRP concentrations in the ATIV, TIV and placebo groups were measured at frequent time points in the first 36 hours post immunisation. Panel (b) PTX3 (no normal range quoted) concentrations on Days 0–5, including frequent time points in the first 36 hours post immunisation are shown for the ATIV, TIV and placebo groups. Ribbons indicate group SEM. The vertical red line indicates the time of discharge from the residential unit.

During the inpatient stay, a clear (~5-fold) increase in the levels of C-reactive protein (CRP; a biomarker of acute inflammation) was only seen in the ATIV group (Fig. 3a). These levels began to rise above baseline from 16 hours post immunization and peaked at 48 hours, but remained below the range associated with clinically relevant inflammation. No changes were observed in any other group.

In Study A, levels of pentraxin 3 (PTX3; Fig. 3b), a protein related to CRP which also plays a role in the innate response^18^, showed that there was more background variability in the assay. Still, a relatively modest (~30%) increase could be seen 24 hours after immunization with ATIV. No clear response was seen with the other vaccines.

**Figure 4:**
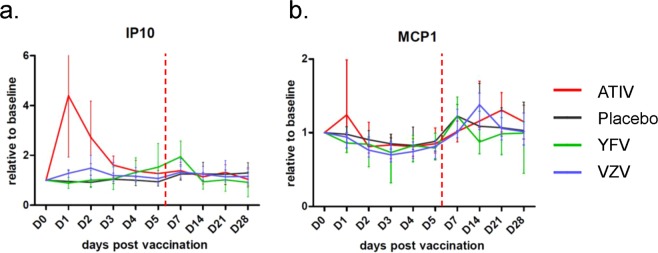
Changes in serum IP-10 and MCP-1 concentrations post immunization. Levels of interferon γ-induced protein 10 (IP-10; panel a) and monocyte chemoattractant protein 1 (MCP1; panel b) were measured up to 28 days after immunization on Day (D)0. Values are expressed as group mean fold-change from baseline (D0) with SEM, to accommodate the different absolute concentration ranges. Vertical dotted lines indicate the time of discharge from the residential unit.

Interferons (IFNs) are indispensable for control of viral infections and immune regulation. During the inpatient phase, early serum responses of IFN-γ–induced protein 10 (IP-10/CXCL10) and the interferon-inducible monocyte chemotactic protein 1 (MCP1/CCL2) were only observed for the ATIV and YFV groups. For IP-10, the ATIV elicited robust responses (4 FC from baseline) with high inter-subject variability at D1, while for YFV the levels gradually increased from D3, peaking (at 2 FC) at D7 (Fig. 4a). For MCP-1, low (<1.5 FC) detections were observed in the ATIV group at D1 (Fig. 4b), also with a high degree of variability. After the inpatient phase, low MCP-1 responses (≤1.4 times baseline values) were seen for all vaccine or control groups, suggesting an impact of the changes in trial setting rather than a vaccine-related effect.

### Whole blood transcriptomics

**Figure 5:**
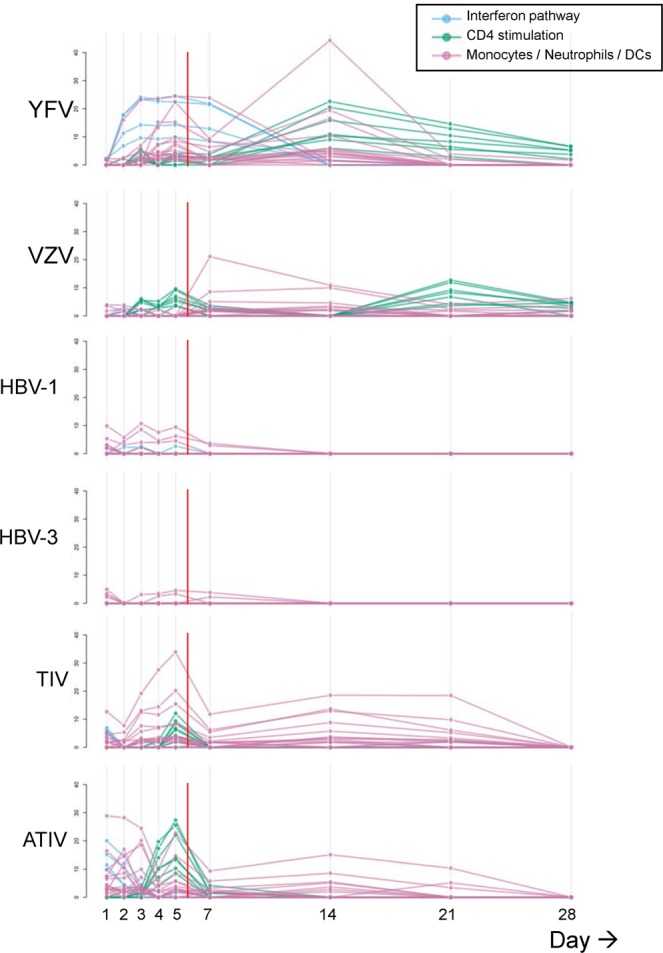
Blood transcriptomic responses for all groups (group level). Peripheral whole blood transcriptomics data are shown by vaccine group. Schematics represent the responses detected for modules of IFN signaling-associated genes, CD4 stimulation-related genes, and innate-cell (monocyte, neutrophil, dendritic cell [DC])-associated genes, as presented by blue, green and pink lines, respectively. Each line represents a single blood transcriptional module (BTM^19,20^). The Y-axis presents -log_10_(FDR) values. Vertical red lines indicate the time of discharge from the residential unit. The contrast tested for a given vaccine and a given time point was the interaction between the differences in expression between this time point and Day 0, and between the given vaccine and placebo.

Peripheral blood transcriptomics data were grouped by previously described functional clusters of blood transcriptional modules (BTMs^19,20^) containing genes regulating IFN-γ, CD4 stimulation or innate cell (monocytes, neutrophils, DCs)-associated responses (Fig. 5). As before, there were significant differences between the inpatient and outpatient time points (at false discovery rate [FDR]-adjusted *P-*value < 0.05), especially in the expression of globin genes (indicative of different blood cell frequencies). As this was not consistently seen across all BTMs and vaccine groups, these differences were most likely associated with the specific kinetics of the different phases of the innate and early adaptive response. The data also revealed distinctly different kinetic patterns between live and subunit vaccines, and allowed discrimination based on the participants’ priming statuses (for both HBV groups) and on the vaccine adjuvantation (for the ATIV and TIV groups).

In Study A, the (previously immunologically naïve) YFV group exhibited a gradual increase in innate cell- and IFN-associated transcripts in the first week. This was followed, between D2 and D28, by gene responses involved in CD4 stimulation, which peaked at D14. The early IFN-associated response was the main discriminant between the two live vaccine groups. Indeed, in the VZV vaccine recipients, who were all seropositive for the virus, activation of CD4-stimulating genes between D2–D5 was followed by responses of innate cell-associated transcripts at D7 and D14, and then by a second activation of adaptive cell-related transcripts at D21. These patterns were associated with differences in the vaccine composition and in the participants’ immune statuses.

In contrast to the live vaccines, the HBV vaccine in Study B elicited in both naive and primed subjects only modest innate cell-related responses, which were seen in the first week and had contracted to baseline at D14. In addition, a low-level activation of IFN-related responses was detected, but only in the naive subjects of the HBV-1 group. No activation of CD4 stimulation-related genes was observed in either group of subjects.

In Study C, both groups of influenza vaccine recipients exhibited similar kinetic patterns, showing early peaks of IFN-related and innate cell stimulation-associated genes at D1. This was followed by CD4-stimulating responses peaking at D5, with earlier detections and higher-level responses for the ATIV group. The latter observation was most likely associated with the presence of the MF59 adjuvant. A second peak of innate cell-associated genes was detected at D5, and these responses were in both groups sustained up to D21.

**Figure 6:**
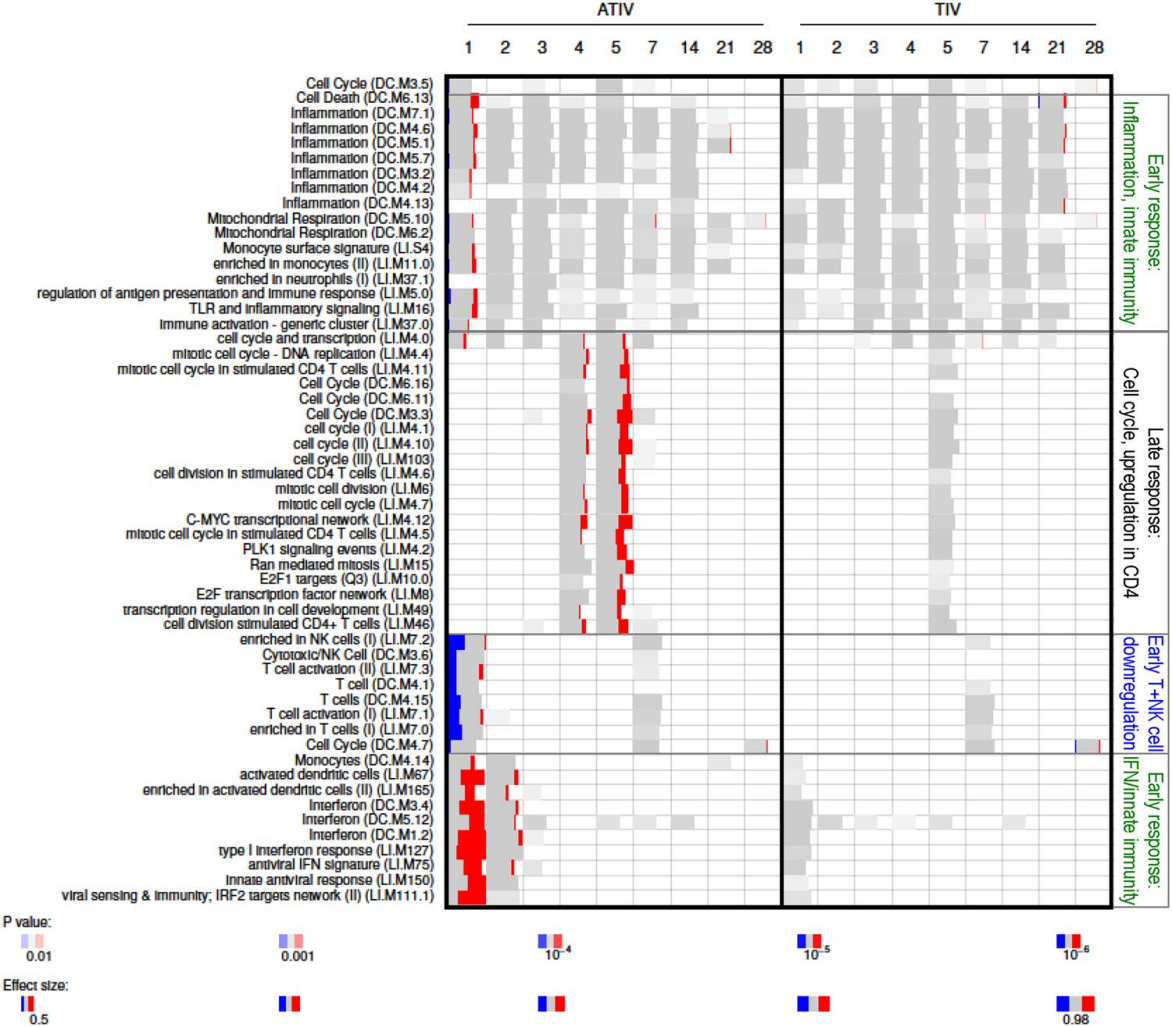
Blood transcriptomic responses for the TIV and ATIV groups in Study C. Heatmap presentations of responses for the individual BTMs of subjects vaccinated with TIV and ATIV are shown. Numbers shown above the heatmap represent the time-points post vaccination, expressed in days.

More detailed inspection of the activated BTMs in Study C revealed similar functional patterns between both groups, but with marked, statistically significant inter-group differences in response levels (Fig. 6). Indeed, while only low-level signals were detected after TIV administration, with significant responses for only a few modules, higher-level responses were seen in the ATIV group. Responses included upregulation of modules relating to innate immunity and IFN-related responses, and downregulated T-cell activation-associated modules, both observed at D1. This was followed by upregulation of late-phase response modules governing CD4, C-MYC and PLK1 signaling, seen between D4 and D6 (at FDR-adjusted *P-*value < 10^−4^). In both groups, very low responses for genes associated with monocytes and T cells were already detected at D0 (at FDR-adjusted *P-*value < 0.05), however the effect size was small (area under the curve [AUC] < 0.65), and there were no associated genes with significant differences in expression).

**Figure 7:**
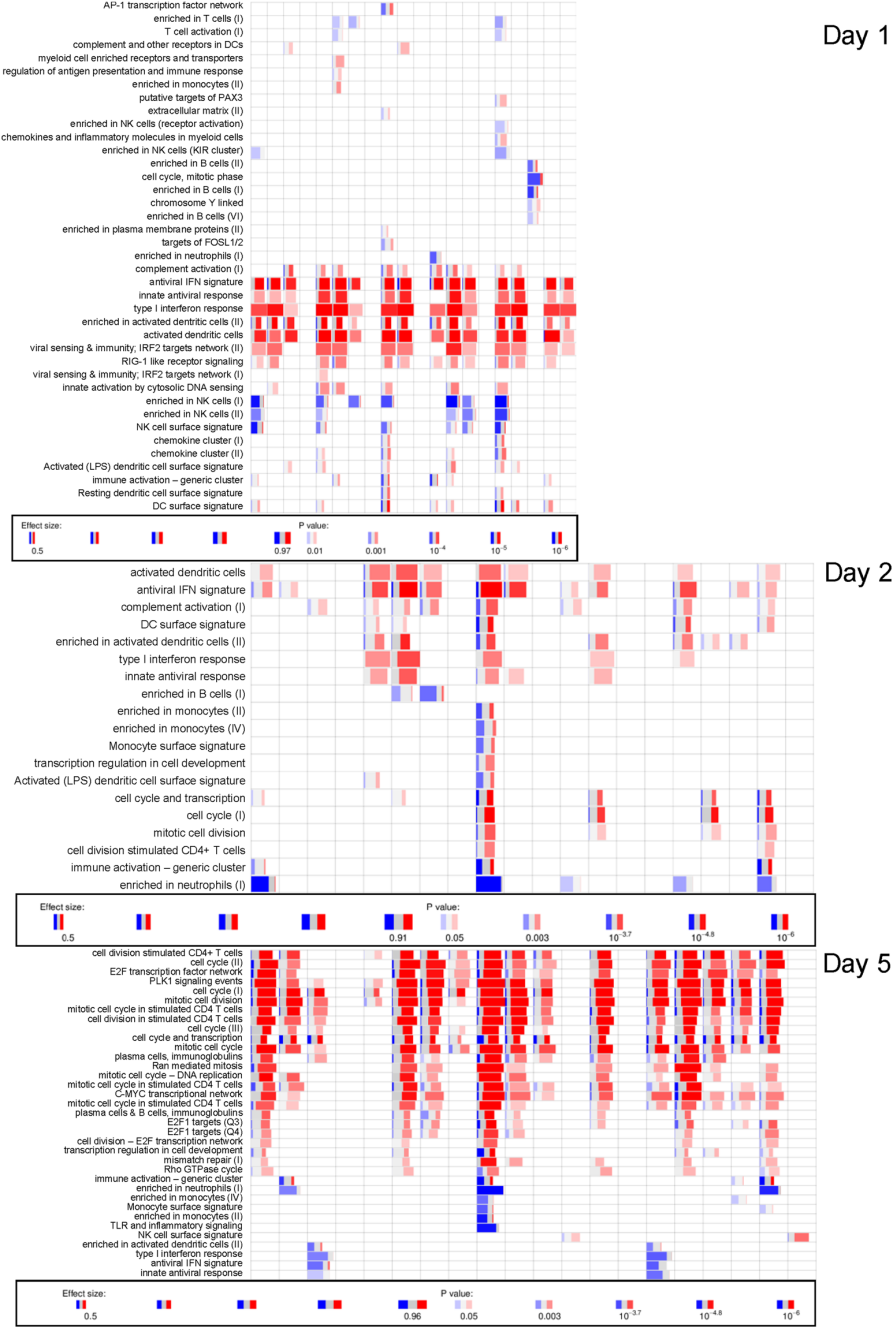
Interindividual variability in transcriptomics profiles of the ATIV group. Legend as for Fig. 6. Each column corresponds to an individual subject (presented in the same sequence across the three days).

Inter-subject variability in gene expression was observed in all vaccine groups, as illustrated by the responses in the individual ATIV recipients (Fig. 7). We also sought to determine whether the high degree of variability in IFN-related protein responses in serum (see Fig. 4) was correlated to inter-subject variability in gene responses in this group. The data revealed substantial inter-individual variability in gene response profiles. Focusing on the IFN-associated modules, we observed that 5, 6 and 4 of the 20 subjects did not respond at D1, D2 and D5, respectively. Most of these non-responders did respond at one or two of these time-points, and one subject did not respond at any of them. Such between-subject variability should be considered in future confirmatory studies. Ideally, this should be compensated for by increasing the sample size, and be accounted for in the data analysis.

**Figure 8:**
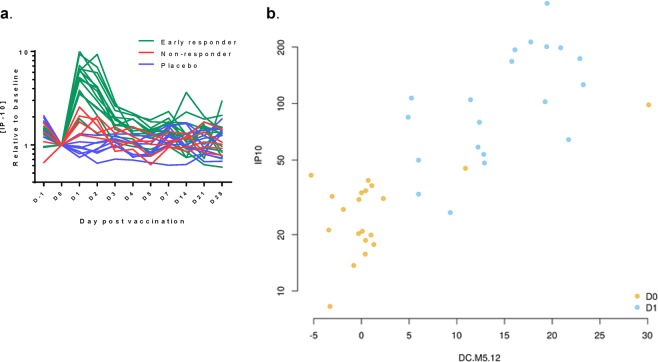
Correlation between serum IP-10 levels and IFN-related gene activation. (**a**) ATIV recipients were stratified into ‘early responders’ or ‘non-responders’ with respect to the presence or absence of early (Day [D]1) enrichment in the IFN signatures. Serum IP-10 levels categorized by responder status are presented for the ATIV and placebo groups. (**b)** Eigengene plot showing the correlation between the eigengene of the ‘Interferon’ blood transcriptional module (BTM) DC.M5.12^20^ and the expression of IP-10 in the ATIV group at D0 and D1. Eigengene (as defined previously^45^) of a set of genes is calculated based on the expression of all genes in a gene set (BTM) and is correlated with the expression of the majority of the genes in the BTM. Pearson’s r = 0.81; p < 1e-6.

Further individual analysis of the relationships between the D1 IFN signatures and serum IP-10 responses was done by stratification of the IP-10 levels by the subject-matched ‘early transcriptomic responder’ status. The data revealed a clear link between these two parameters (Fig. 8a), which was confirmed when we plotted the IP-10 levels at D0 (baseline) and D1 against enrichment levels for one specific IFN-related module (DC.M5.12^20^; Fig. 8b).

## Discussion

Using licensed safe antiviral vaccines as benchmarks, we undertook linked, residential clinical studies to generate a data-set of vaccine-elicited responses up to one month post-vaccination. The data set contained clinical events as well as responses at the level of gene transcripts. Previously, we described some highly specific aspects of the safety and adaptive immune response evaluations for some of these trials^15–17^. Here, we report the reactogenicity and early vaccine response data. The data suggest that the inflammatory response, with respect to physiological parameters, serum markers and innate signaling transcripts, is a function of both the vaccine composition (live *vs*. inactivated; nonadjuvanted *vs*. adjuvanted) and the presence of immune (CD4 T-cell or B-cell) memory in the recipient.

As a first approach, we assessed the impact of immune memory after the low-level stimulation by the alum-adjuvanted recombinant subunit HBV vaccine, for which 3 doses are required to obtain protective responses^21^. The vaccine elicited low-level gene responses, which lacked CD4 activation-related transcripts in both naive and primed subjects, and IFN-related transcripts in primed subjects. In contrast to the gene responses, no differences between the first or third dose were detected with respect to inflammatory responses (CRP, innate cells), vital signs or adverse event reactogenicity (or lack thereof). This suggests that aluminum salt is a poor activator of immune transcripts, which is consistent with other studies^4,22,23^. Furthermore, this suggests that the expression of transcripts depends on the individual’s pre-existing status of immune priming. Unfortunately, this study could not discriminate mechanistically between the immune system components that were responsible for the differences between naïve and primed individuals, as no studies on blood cell populations were performed.

In parallel, we assessed the impact of the o/w adjuvant MF59, using a subunit vaccine type administered to subjects with presumably comparable immune statuses at baseline. Aligned with the potent activation of innate immunity reported for MF59^24–26^, the ATIV induced the most prominent innate response. This response was characterized by a sequential increase in mean body temperature and lack of expected decrease in mean heart rate in the first night. This was followed at D1 by activation of IFN-related and innate cell-related genes, by increased monocyte and neutrophil counts (coinciding with decreased lymphocyte counts), and by increased PTX3, serum IP-10 and MCP-1 protein responses, and then by a CRP response at D2. The observed serum PTX3 response is consistent with the increased PTX3 expression seen in the muscles of MF59-injected mice, and maybe also with the fact that PTX3 can act as an endogenous adjuvant of certain antimicrobial responses^26,27^. These data aligned with other evaluations of both MF59-containing influenza vaccines and conventional subunit TIVs^26,28–30^. They are also consistent with clinical data for vaccines containing AS03, an o/w adjuvant that contains α-tocopherol^22,31^. Notwithstanding the evident quantitative differences in transcriptomic responses (Fig. 6b), the signatures of these responses were qualitatively similar for both vaccines. These signatures were characterized by a sharp peak around D1, when monocyte and neutrophil counts increased, and a prominent role for genes associated with early IFN signalling as well as for antigen-processing and presentation-related genes. Interestingly, the concurrent increase in peripheral myeloid cells and blood depletion of lymphoid cells, as seen at D1–2 (a phenomenon also seen with AS03-adjuvanted HBV vaccine^22^) may reflect local recruitment of lymphoid cells. However, this would need to be confirmed by direct investigation of the events in tissue at the sites of the immune response.

Though the majority of the ATIV recipients exhibited robust transcriptomic responses, the high level of inter-subject variability in transcriptomic responses was remarkable, as observed for example in the interferon modules. This heterogeneity may be explained by the subjects’ variable degrees of immune memory derived from previous infection and/or vaccinations with different matching or non-matching strains (which we did not investigate), in combination with the small sample size (since heterogeneity was also observed for the other vaccines). It is also possible that the gene-set enrichment analysis based on absolute values of gene expression (rather than on fold-changes) was not a sufficiently sensitive method. While variability in individual measurements of gene expression may also be due to technical variability (which could have prevented detection of enrichment in some cases), this is unlikely to have played a significant role, given the remarkable uniformity of the analyzed control RNA samples. In any case, the variability in gene expression observed in some ATIV recipients over time did not appear to have affected their ability to mount strong influenza-specific antibody and cellular responses, as previously reported^15^.

Preclinical data suggest a link between o/w-adjuvant-mediated activation of innate immunity and reactogenicity, which is corroborated by clinical data (reviewed in ref. ^32^). Indeed, for an AS03-adjuvanted pandemic influenza vaccine, there was a trend for an association between the occurrence of medium/high-level reactogenicity and increased IP-10 levels, but no such putative link was seen for CRP or other inflammatory markers in serum^3^. In the current study, individual analysis detected a relationship between the D1 IFN signatures and serum IP-10 responses, however it remains to be determined to which extent these immune phenotypes correspond with reactogenicity for this vaccine, and whether such links are translatable to other vaccines and/or adjuvants.

Pre-vaccination signals for monocytes-related and T-cell-related modules were weak for both influenza vaccines, and were shown to be enhanced after vaccination. In the abovementioned study with the AS03-adjuvanted pandemic A/H1N1 vaccine^3^, the subjects experiencing medium/high-level reactogenicity shared a baseline gene expression associated with certain B-cell phenotypes, suggesting that transcriptomic changes before vaccination might be predictive of clinical events post-vaccination. An impact of the baseline transcriptome on the innate vaccine responses has also been reported for the YFV and HBV vaccines^33,34^, and the links with reactogenicity warrant further investigation. Pre-vaccination signals will be evaluated in-depth in the forthcoming larger clinical trials using the adjuvanted influenza vaccine.

Finally, we evaluated two different live-attenuated vaccines (inducing variable levels of pathogen-associated molecular patterns-mediated innate immunity), in the absence (YFV) or presence (VZV) of immune memory. Although based on very different viruses, both vaccines exhibited a gradual and (as compared with subunit vaccines) suspended increase in gene responses. Due to the kinetics of viral replication, peaks were detected around D5–D7, around the same time as the monocyte response peaked in blood. The YFV vaccine, a potent inducer of innate immunity, can elicit long-term activation of ‘trained’ monocytes and NK cells, which is for some activation markers detectable for up to 2 months post-vaccination^34–37^. This aligns with the readily detectable innate gene expression and with the early serum IP-10 and CRP responses observed here, in the absence of immune memory. By contrast, the lack of significant activation of IFN-associated genes by the VZV vaccine may be linked to the pre-existing antibodies in these subjects (which may have reduced viral replication), and to the higher level of vaccine attenuation as compared to the YFV vaccine. Indeed, previous data demonstrated that even with a higher-dosed VZV vaccine as the one used here, the induced transcriptomic responses remained relatively low^38^.

Some strengths and intrinsic limitations of the present study should be highlighted. On the one hand, the controlled inpatient setting employed in this study enabled detection of subtle vaccine-specific changes in vital signs in the first night post-vaccination, that would have gone undetected in conventional trials. On the other hand, this setting also entailed the risk of an ‘incarceration’ effect, with the consequent deviations of physiological/laboratory safety parameters from their individual ‘normal’ values. The placebo-controlled study design was therefore vital to discriminate between such an effect and any vaccination-related impacts on inflammatory parameters. Interestingly, the incarceration effect seen in the blood chemistry was not mirrored by the CRP, chemokine or transcriptomic responses. This might be explained by the transient nature of the latter responses, peaking in the first few days, or by the assumption that these changes only manifest locally, due to low-level spillover from the draining lymph node into the serum. A limitation was that these small-scale observational studies with only exploratory endpoints did not allow comparative analyses to be done between groups, or definitive conclusions on biomarkers to be drawn. They also did not allow analyses on the effects of any factors potentially affecting innate immunity and reactogenicity, such as ethnicity^16^, age (even within this population of healthy adults^3^), or gender^39^. These factors would also need to be considered for development of predictive personalized biomarkers of vaccine safety.

In conclusion, the current data informed the design of subsequent research phases. Based on its distinct physiological and immunological response patterns, the ATIV was selected (together with other vaccines not evaluated here) for further studies in confirmatory Phase IV clinical trials, and in human muscle biopsy studies. This research aimed to associate the transcriptomic changes in peripheral blood with those in sorted cell populations, and to dissect out the local responses underlying differential reactogenicity to different adjuvants. These results are supported by parallel preclinical BIOVACSAFE studies on adjuvants, as done for alum and MF59 in mice^7,10^. The collected data will be subjected to integrated systems biology studies, to identify the biomarkers that could accelerate development of safer and more effective new vaccines.

## Materials and Methods

### Study design

Studies A, B and C (ClinicalTrials.gov: NCT01765413, NCT01771354 and NCT01771367, respectively) were subject/laboratory-blinded, randomized, placebo-controlled studies, conducted from February 2013 through September 2014 at the University of Surrey Clinical Research Centre (Surrey, Guildford, UK) in accordance with the Helsinki Declaration and Good Clinical Practices. Protocols (nos. CRC305A-C) and CONSORT checklists were published previously^15–17^. Ethical approvals were obtained from the NRES Committee London - Surrey Borders (nos.: 12/LO/1871, 12/LO/1899, 13/LO/0044 respectively). All participants provided written informed consent prior to enrolment. Blood collection for biochemistry, hematology, whole blood transcriptomics and immunology parameters, and monitoring of reactogenicity and vital signs, was performed at scheduled time-points before and after immunization (Fig. S1).

### Objectives

The study objective was to generate an exploratory ‘training’ set of data characterising reactogenicity as well as physiological and innate immune responses following immunisation with licensed vaccines. This data set would then be used to identify putative biomarkers of vaccine reactogenicity in subsequent research phases, that could guide the design of follow-on large-scale non-residential confirmatory studies, using selected vaccines and time points.

### Participants and demographics

Healthy male and female volunteers 18–45 years of age were enrolled and randomized into two (Study B) or three (Studies A and C) study groups (Fig. S3). Each subject acted as their own control for kinetics with comparison from the baseline preimmunization levels of the measured biomarkers.

A total of 49, 25, and 49 healthy adults enrolled in Studies A, B and C, respectively, were vaccinated, and all but two subjects completed the respective studies (Table 1 and Fig. S3). These two subjects (one each in the VZV or HBV-3 groups) withdrew consent not due to an AE, either during or after the inpatient phase, and were excluded from the analyses. Demographic profiles were broadly balanced between the groups in terms of mean age and ethnicity, but there was a male preponderance in the YFV, VZV and HBV groups. No YFV viremia was detected in the recipients of live YFV vaccine, at any time point.

### Vaccinations

Participants were admitted to the research center on D-1 to acclimatize and for pre-immunization blood sampling. All blood samples were taken within 15 minutes of the designated time. All treatments were administered into the deltoid muscle or overlying subcutaneous tissue at 08:00 h (±15 minutes). All participants received only a single injection when immunized with either a single vaccine or a placebo, according to the group allocation. In the vaccine groups in Study A, participants seropositive for anti-VZV antibodies (as confirmed by serology and immunization history) received live-attenuated VZV (Oka strain) vaccine (Varilrix supplied as 10^3.3^ plaque-forming units; GSK), while participants seronegative for anti-YFV antibodies and without a prior history of YFV vaccination received live-attenuated YFV vaccine (Stamaril containing ≥ 1000 LD_50_ units; 17 D-204 strain; Sanofi Pasteur). Treatments were administered subcutaneously (deltoid region) as a single 0.5 mL dose, delivered at D0. In Study B, participants seronegative for anti-HBV antibodies and without a prior history of HBV vaccination received intramuscular alum-adjuvanted (rDNA) HBV vaccine (Engerix-B1 containing 20 μg adsorbed HBsAg, GSK). The HBV-1 group received a priming dose at D0. A second HBV dose was administered as an outpatient vaccination at D28, and then the same participants were re-admitted to the research center on D168 and given a third dose at D169 (designated group HBV-3). In Study C, participants assumed to be primed with hemagglutinin via vaccination or natural infection, received a single 0.5 mL IM dose of either non-adjuvanted or MF59-adjuvanted seasonal trivalent influenza surface antigen (inactivated) vaccine (TIV - Agrippal or ATIV - Fluad, respectively; Novartis Vaccines) at D0. Both vaccines contained the recommended composition for the 2012/2013 northern hemisphere influenza season^15^. The control groups included in each study received saline placebo intramuscularly (groups B and C) or subcutaneously (group A) at the same time points as the vaccinees. Placebo subjects were later pooled to one group (N = 20), excluding the data from D167 onwards for the control group for HBV-3 (N = 4), which were analyzed separately (see Fig. S3).

In the inpatient phase, participants were admitted to the chronobiology ward of the Surrey Clinical Research Center at D-1 where they remained for 7 days and 6 nights, and allowed home after the 20:00 h blood draw on D5. While inpatients, they were held to controlled diet, exercise and sleep routines (lights on/off at 07:30 h/22:30 h; meals provided at 09:30 h/13:15 h/18:30 h; snacks provided at 10:45 h/21:15 h; fluids provided *ad libitum*; strenuous activities, alcohol, caffeinated drinks and tobacco-containing products were prohibited). Time-points of sample collection, and of the clinical events monitoring conducted during the inpatient phase and at the outpatient follow-up visits (at D7, D14, D21 and D28) are presented in Fig. S1.

### Reactogenicity

Reactogenicity (local and systemic unsolicited AEs, classified using the medDRA-preferred terms) were recorded throughout the study. Participants were queried for any AEs on a daily basis during the inpatient stay and at each outpatient visit, but there were no symptoms of interest specifically solicited. Local complications from indwelling cannulas such as phlebitis were also checked for daily and recorded, but were excluded from reactogenicity calculations. Laboratory AEs were also excluded from reactogenicity calculations. To obtain a uniform reactogenicity quantification across the groups, that could serve as a basis for the correlative analyses in the subsequent studies, AEs were first classified by the clinicians as either related or unrelated to immunization, and only the AEs that were considered as related were included in the calculation. Each AE was then described by the participant, or was measured by the investigators (in the case of local inflammatory reactions). AEs were quantified by referencing to the appropriate FDA tables (*Guidance for Industry - Toxicity Grading Scale for Healthy Adult and Adolescent Volunteers Enrolled in Preventive Vaccine Clinical Trials*, FDA, Sept. 2007) adapted to the protocol, and were graded as mild (no interference with activities), moderate (some interference) or severe (significant interference), or they were quantified using the measurement scale provided for local injection site reactions. The standardized grading was then translated to a numerical value on a 1, 2, 3 scale, and multiplied by the AE duration (expressed in days), to generate a reactogenicity score. For each vaccine group, the number of treatment-related AEs with an onset between D0 and D7 post-immunization, sum of the associated reactogenicity scores (the vaccine “reactosum”), proportion of participants recording any treatment-related AE with an onset between D0 and D7 post-immunization, and the mean participant reactosum, were calculated.

### Physiological and laboratory assessments

Vital signs (heart rate, oral temperature, and diastolic/systolic blood pressure measured following five minutes in a supine position were recorded four-hourly (±15 minutes) on D0–3, and twelve-hourly on D4–5 of the inpatient stay and at outpatient visits. Standard laboratory blood chemistry (liver, renal and bone panels), CRP, and hematology (automated Full Blood Count with white cell, erythrocyte sedimentation rate) assays were performed as described^16^. They were performed once daily at D-1 ─ D5, and at each outpatient visit (Fig. 1). CRP concentrations were assayed in a two-step process: initially a standard sensitivity assay was used, and samples with a value < 10 mg/L were then re-assayed using a highly sensitive CRP assay, and this value alone was reported. In a subset of participants (ATIV, TIV and placebo groups in Study A; N = 20, 21 and 8, respectively), serum CRP and PTX3 concentrations were measured in stored serum samples at more frequent time points, using for PTX3 an in-house ELISA assay, as described previously^40^.

### Cytokine/chemokine measurements

Blood samples for assessment of inflammatory mediators (chemokines, cytokines) in serum, and of whole blood gene expression were collected before and after vaccination (Fig. S1). Serum cytokine and chemokine concentrations were measured on a Luminex platform with custom human cytokine kits from R&D (R&D Systems GmbH, Wiesbaden, Germany) and Millipore (Merck Chemicals GmbH, Darmastadt, Germany) according to the manufacturer’s instructions. The following analytes were examined: TNF-α, IL-8, MCP1, IFN-γ, MIP-1α, IL-1α, GM-CSF, IP-10, TNF-RR1, IL-6, VEGF, PTX3, IL-2, IL-5, IL-2Ra (R&D panel), and IFN-α2, IL-12p40 and IL-1RA (Millipore panel). In addition to the standards provided with the kits, biological reference reagents from NIBSC (kindly provided by Dr. Mei Mei Ho) were included to check for inter-assay variability. Analytes CCL5 and TREM1 were quantified with DuoSet ELISA kits from R&D.

### Gene expression profiles

#### Microarray analysis

Peripheral blood was drawn into PAXgene tubes (PreAnalytiX) and RNA was extracted on the automated QIAcube system (Qiagen) using the PAXgene Blood RNA kit (Qiagen) according to the manufacturer’s instructions. Quality control and quantification of isolated RNA was analyzed with an Agilent 2100 Bioanalyzer (Agilent Technologies) and a NanoDrop 1000 UV-Vis spectrophotometer (Thermo Fisher Scientific). Microarray experiments were performed as single-color hybridization, and RNA was labeled with the Low Input Quick-Amp Labeling Kit (Agilent Technologies). In brief, mRNA was reverse transcribed and amplified using an oligo-dT-T7 promoter primer, and labeled with cyanine 3-CTP by T7 *in vitro* transcription. After precipitation, purification and quantification, 0.75 μg labeled cRNA was fragmented and hybridized to custom whole genome human 8 × 60 K multipack microarrays (Agilent-048908) according to the supplier’s protocol (Agilent Technologies). Scanning of microarrays was performed with 3 μm resolution and 20-bit image depth, using a G2565CA high-resolution laser microarray scanner (Agilent Technologies). Microarray image data were processed with the Image Analysis/Feature Extraction software G2567AA v. A.11.5.1.1 (Agilent Technologies), using default settings and the GE1_1105_Oct12 extraction protocol.

#### Microarray normalization and quality control

Blinded primary readouts of the microarrays were read, background corrected, normalized and controlled for quality using the R package limma^41^ (version 3.30). For background correction, the gProcessedSignal from the primary readouts was used. Between-array normalization was done using the quantile method in limma. Quality control relied on density plots, testing for outliers, visualization with principal component analysis and visual inspection of individual array images. The normalized data was locked and submitted to project management. Next, the data was unblinded for further analysis. All primary readouts and the background corrected and normalized data are available from the Gene Expression Omnibus (GEO) database under the BioProject identifier PRJNA515032 (https://www.ncbi.nlm.nih.gov/bioproject/?term=PRJNA515032).

#### Differential gene expression analysis

Prior to the analysis, the hybridization control samples were removed from the data set, and the gene expression values were averaged for each probe over all replicates of that probe on the microarray, using the ‘avereps’ function from limma. Differences in gene expression for each vaccine at each time point tested were assessed using a three-factor linear model in limma. The expression was fit to time point, group (vaccine vs placebo) and subject. The contrast tested for a given vaccine and a given time point was the interaction between the difference in expression between this time point and the D0 time point, and the difference between the given vaccine and placebo, as follows: (V_Dn_ − V_D0_) − (P_Dn_ − P_D0_), where V stands for the given vaccine, P stands for placebo, Dn stands for the given time point, and D0 stands for the sample collected at vaccination. The p-values were corrected for false discovery rate using the Benjamini and Hochberg (BH) procedure^42^.

#### Gene set enrichment analysis

Gene set enrichment was tested with the CERNO algorithm implemented in the R package tmod^43^, version 0.40, with the MSD metric for ordering the genes^44^. For testing, the gene sets (BTMs) defined by Li *et al*.^19^ and Chaussabel *et al*.^20^ were used. P-values were corrected using the BH procedure; gene set enrichments with q < 0.05 were considered significant. Enrichment was visualized with the tmodPanelPlot function from tmod.

#### Analysis of individual variability

For each individual at each timepoint, the genes were ordered by the measured expression, and the gene set enrichment was performed on the ordered list of genes, using CERNO statistic as implemented in tmod.

#### Correlation analysis

To test for enrichment in genes correlated with a given cytokine response, the following procedure was applied. First, for the given parameter, the Spearman correlation coefficient between this parameter and the expression of each gene was calculated. Next, the genes were ordered by the decreasing absolute correlation coefficient, and the CERNO enrichment test was applied to the ordered list of genes.

#### Statistical methods

Safety and immunology parameters were reported descriptively and tabulated as SEM bars/ribbons. Computations were performed in R software (https://www.r-project.org/).

### Previous presentations

The current research work was presented in part at the ‘World Vaccine Congress’, Washington DC, USA, April 2–5, 2018. The present manuscript has not been submitted elsewhere.

### Clinical trial registration

Clinicaltrials.gov registration:

305A: NCT01765413, 15/11/2012

305B: NCT01771354, 15/11/2012

305C: NCT01771367, 15/01/2013

### Trademarks

Varilrix and Engerix are trademarks owned by or licensed to the GSK group of companies. Stamaril is a trademark of the Sanofi-Pasteur group of companies. Fluad and Agrippal are a trademark of Seqirus, part of the CSL group of companies.

## Supplementary information

Supplementary Material.
