# Supplementary material for: Characterization of potential biomarkers of reactogenicity of licensed antiviral vaccines: randomized controlled clinical trials conducted by the BIOVACSAFE consortium
Source: Sci Rep. 2019 Dec 30;9:20362. doi: 10.1038/s41598-019-56994-8 (PMC6937244; doi:10.1038/s41598-019-56994-8)
Supplement: Supplementary file 1 — Supplementary Material. [file 41598_2019_56994_MOESM1_ESM.pdf]

Characterization of potential biomarkers of reactogenicity of licensed antiviral vaccines: randomized controlled clinical trials conducted by the BIOVACSAFE consortium

Supplementary Material

January Weiner[a*], David JM Lewis[b*], Jeroen Maertzdorf[a], Hans-Joachim Mollenkopf[a], Caroline Bodinham[b], Kat Pizzoferro[b], Catherine Linley[b], Aldona Greenwood[b], Alberto Mantovani[c,d,e], Barbara Bottazzi[c], Philippe Denoel[f], Geert Leroux-Roels[g], Kent E. Kester[h], Ingileif Jonsdottir[i], Robert van den Berg[j], Stefan H.E. Kaufmann[a], Giuseppe Del Giudice[k§]

[a]Max Planck Institute for Infection Biology (MPIIB), Department for Immunology, Berlin, Germany
[b]Surrey Clinical Research Centre, University of Surrey, Guildford, UK
[c]Humanitas Clinical and Research Center-IRCCS, Rozzano, Italy
[d]Humanitas University, Pieve Emaneuele, Italy
[e]The William Harvey Research Institute, Queen Mary University of London, London, UK
[f]GSK, Rixensart, Belgium
[g]Center for Vaccinology, Ghent University and University Hospital, Ghent, Belgium
[h]Sanofi-Pasteur, Swiftwater, PA, USA
[i]deCODE genetics/Amgen Inc., Reykjavik, Iceland
[j]GSK, Rockville, MD; USA
[k]GSK, Siena, Italy
*These authors contributed equally

[§]**Corresponding author**: Giuseppe Del Giudice, email: giuseppe.x.del-giudice@gsk.com

Figure S1. Study design. Screening procedures included written informed consent, physical examinations, as well as recording of demographic data, medical history, vital signs (heart rate, blood pressure, oral temperature), laboratory safety data, pregnancy tests (female participants), and blood sampling for VZV/YFV serology in Study A. In study B, participants received their first dose of HBV vaccine or placebo at Day (D)0 (HBV-1), then a second dose at D28 during the outpatient follow-up, and a third dose at D169 during second inpatient stay (HBV-3). Due to the large numbers of individual samples generated all collected samples were biobanked. Gene expression was assessed based on RNA analysis in whole blood. Serum was collected for assessment of immunology parameters including acute phase proteins, cytokines/chemokines and YFV viremia (YFV group; Study A). PBMC data are reported in [15,17]. Adverse event (AE) recording included evaluation of local and systemic AEs.

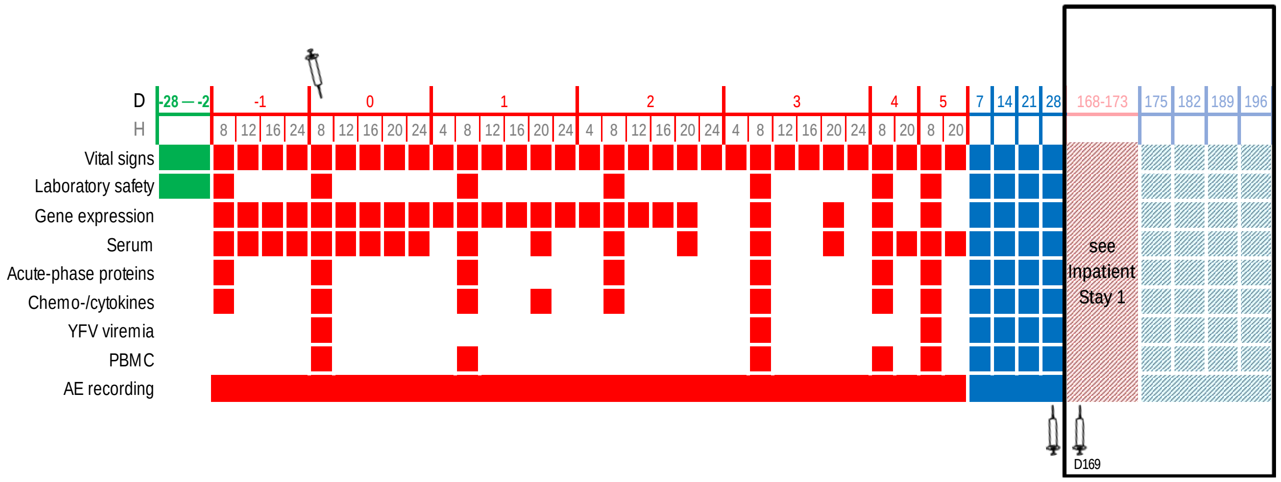

Figure S2: Fold-changes in white blood cell counts after immunization.

The figure shows the mean change from pre-immunisation baseline value (at 08:00 hours on Day 0) in the lymphocyte (red), monocyte (green) and neutrophil (amber) cell count. Values are expressed as fold-change from baseline to accommodate the different absolute count ranges. Ribbons indicate group SEM. Vertical red lines indicate time of discharge from the residential unit.

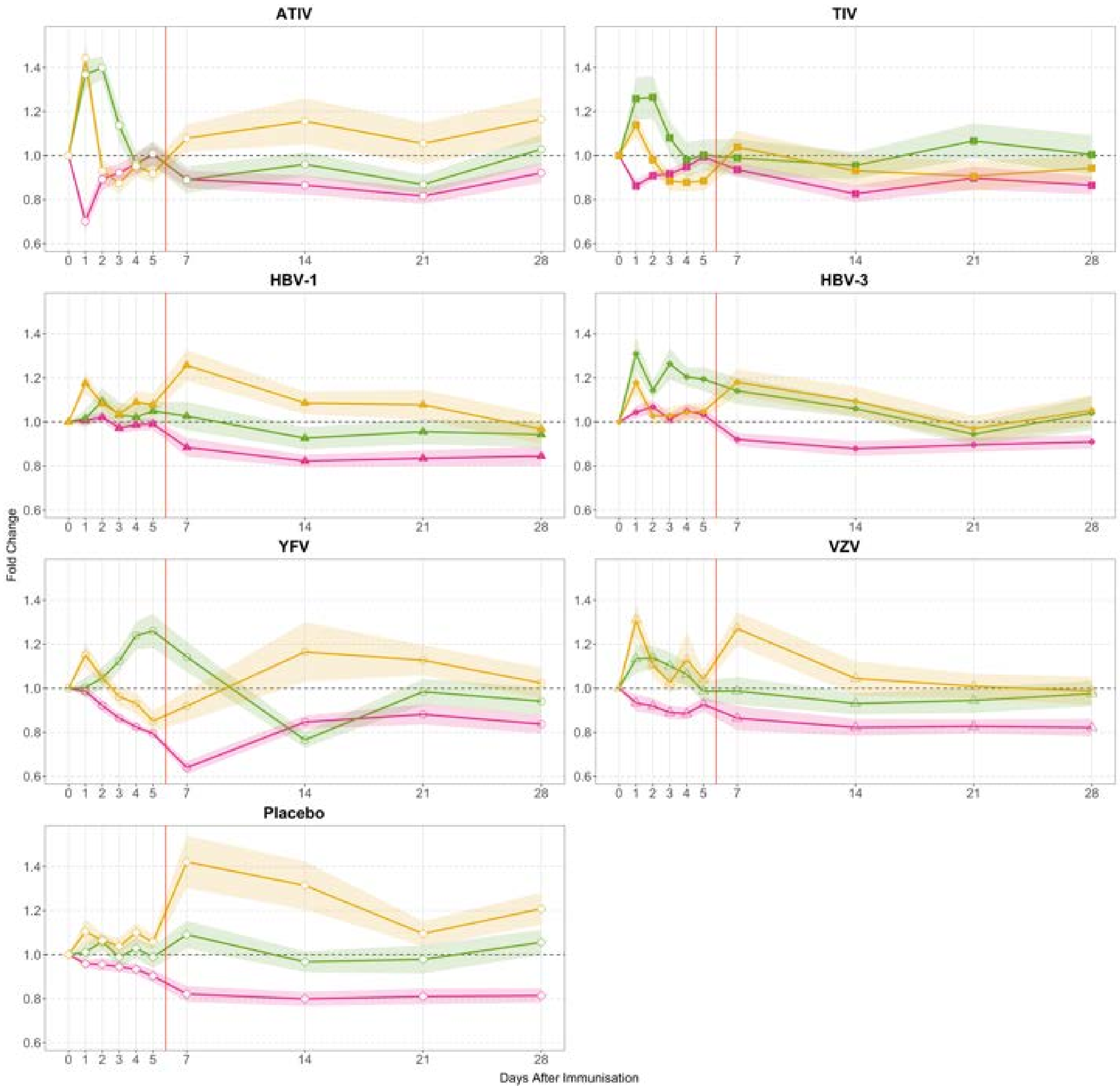

Figure S3. CONSORT participant flowcharts. Diagrams published previously: YFV group [16], TIV and ATIV groups [15,16] and placebo group Study C [15]. The three placebo groups were pooled for the analyses. DI: Discontinued intervention.

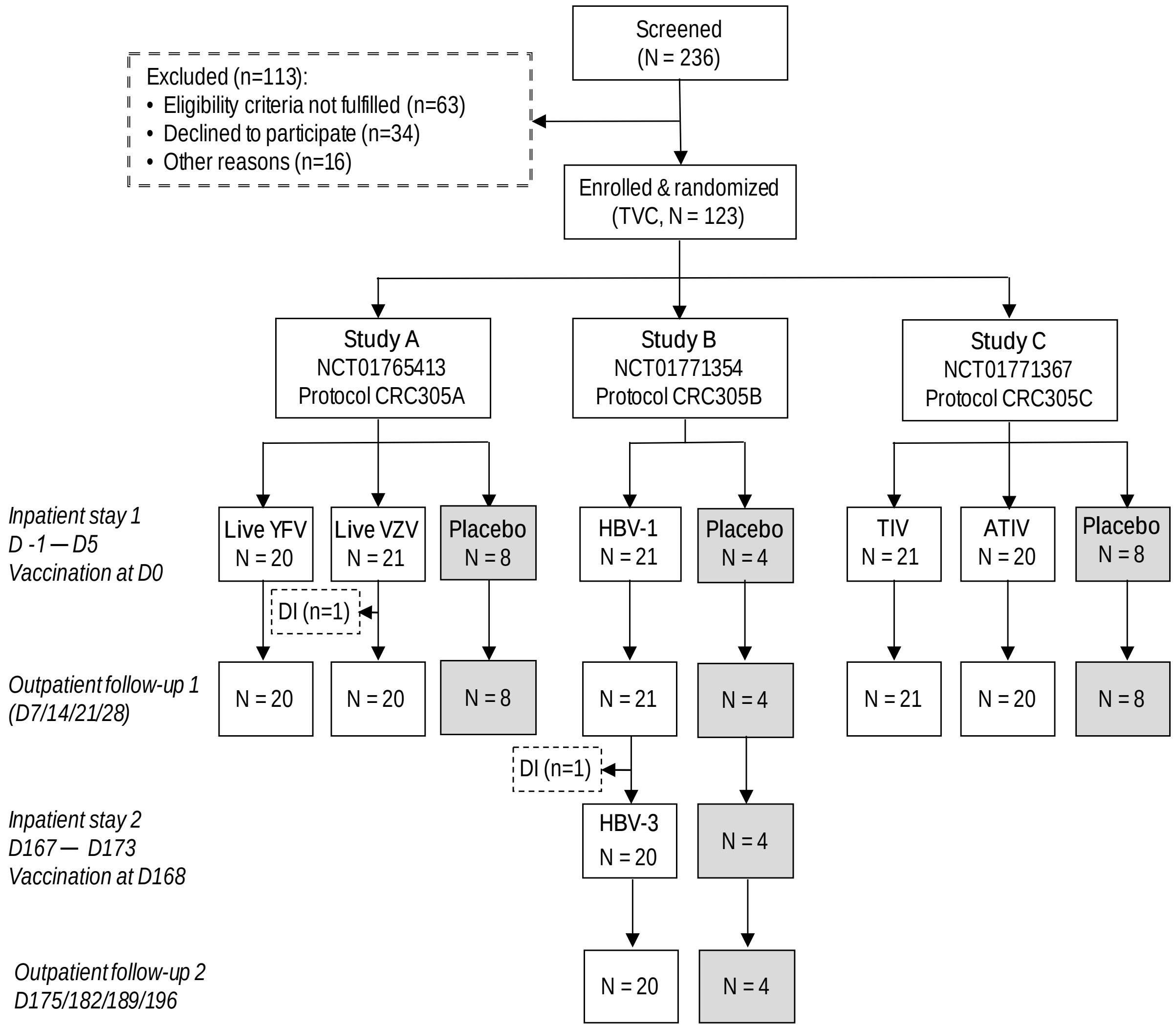

Supplementary Table 1: Changes in serum albumin and uncorrected calcium concentrations post-immunization

Day	Albumin (g/L, NR: 35 – 50)							Uncorrected Calcium (mmol/L, NR: 2.1 - 2.6)						
	ATIV	HBV-1	HBV-3	Placebo	TIV	VZV	YFV	ATIV	HBV-1	HBV-3	Placebo	TIV	VZV	YFV
0	40.7	40.1	38.2	39.1	40.4	39.2	39.2	2.19	2.16	2.13	2.15	2.19	2.18	2.18
1	39.9	39.0	38.2	39.1	39.8	39.1	39.3	2.14	2.17	2.15	2.16	2.16	2.16	2.19
2	39.9	39.9	38.0	39.1	39.8	38.8	38.8	2.14	2.16	2.14	2.15	2.16	2.13	2.16
3	40.2	39.1	38.2	39.3	39.5	39.0	38.8	2.14	2.17	2.13	2.17	2.15	2.16	2.16
4	39.9	40.0	38.6	38.5	39.3	39.3	38.9	2.17	2.16	2.16	2.12	2.16	2.16	2.17
5	39.8	40.2	39.3	38.6	39.4	39.6	39.4	2.14	2.19	2.16	2.13	2.17	2.18	2.18
7	43.7	43.0	41.3	43.0	44.0	43.2	42.7	2.28	2.25	2.21	2.25	2.29	2.26	2.22
14	42.9	42.6	42.1	43.2	43.8	42.2	41.5	2.27	2.26	2.23	2.26	2.30	2.27	2.28
21	42.5	43.3	41.9	43.2	43.1	43.4	41.9	2.30	2.26	2.24	2.29	2.29	2.28	2.24
28	43.4	42.3	42.8	42.7	43.0	42.4	42.7	2.27	2.26	2.32	2.29	2.25	2.28	2.30
Day 5-7 Change (%)	9.8	7.0	5.1	11.4	11.7	9.1	8.4	6.3	2.5	2.0	5.8	5.6	3.4	2.0

Mean serum concentrations of albumin and uncorrected calcium at different time points after immunization are shown for all treatment groups together with laboratory normal ranges (NR) and the percentage change in mean values between days 5 and 7 post immunization.
